# Change in emotional self‐concept following socio‐cognitive training relates to structural plasticity of the prefrontal cortex

**DOI:** 10.1002/brb3.940

**Published:** 2018-03-13

**Authors:** Anna‐Lena Lumma, Sofie L. Valk, Anne Böckler, Pascal Vrtička, Tania Singer

**Affiliations:** ^1^ Department of Social Neuroscience Max Planck Institute for Human Cognitive and Brain Sciences Leipzig Germany; ^2^ Department of Psychology and Psychotherapy University of Witten/Herdecke Witten Germany; ^3^ Department of Psychology III University of Würzburg Würzburg Germany

**Keywords:** cortical thickness, emotional word use, meditation, mental training, neuroplasticity, self‐concept content, self‐descriptions

## Abstract

**Introduction:**

Self‐referential processing is a key component of the emotional self‐concept. Previous studies have shown that emotional self‐referential processing is related to structure and function of cortical midline areas such as medial prefrontal cortex (mPFC), and that it can be altered on a behavioral level by specific mental training practices. However, it remains unknown how behavioral training‐related change in emotional self‐concept content relates to structural plasticity.

**Methods:**

To address this issue, we examined the relationship between training‐induced change in participant's emotional self‐concept measured through emotional word use in the Twenty Statement Test and change in cortical thickness in the context of a large‐scale longitudinal mental training study called the *ReSource Project*.

**Results:**

Based on prior behavioral findings showing increased emotional word use particularly after socio‐cognitive training targeting perspective‐taking capacities, this study extended these results by revealing that individual differences in the degree to which participants changed their emotional self‐concept after training was positively related to cortical thickness change in right mPFC extending to dorsolateral PFC (dlPFC). Furthermore, increased self‐related negative emotional word use after training was positively associated with cortical thickness change in left pars orbitalis and bilateral dlPFC.

**Conclusions:**

Our findings reveal training‐related structural brain change in regions known to be involved in self‐referential processing and cognitive control, and could indicate a relationship between restructuring of the emotional self‐concept content as well as reappraisal of negative aspects and cortical thickness change. As such, our findings can guide the development of psychological interventions targeted to alter specific facets of the self‐concept.

## 1 INTRODUCTION

Advancing the understanding of the self is one of the most challenging endeavors in empirical psychological science with a high relevance for applied domains such as clinical psychology. For example, prior research showed that alterations of the emotional facets of the self are associated with maladaptive personality traits including narcissism (Böckler, Sharifi, Kanske, Dziobek, & Singer, 2017; Konrath & Bonadonna, 2014) and with psychopathological conditions such as depression (Nejad, Fossati, & Lemogne, 2013). In the case of depression, there is a strong prevalence of negative self‐focused thinking patterns that form the content of the person's self‐concept and influence his/her mood and behavioral tendencies. Consequently, clinical interventions like cognitive therapy aim at changing the appraisal of negative self‐focused thinking patterns in clinical populations (Hofmann, Asmundson, & Beck, 2013). However, less is known about how targeted mental trainings could induce change in emotional self‐concept content in healthy individuals.

Emotionality is a key element of self‐referential processing. The perceived self‐relevance of information—such as external and internal sensory stimuli, thoughts, and beliefs—depends upon its subjective emotional evaluation and attributed saliency. Converging evidence within the functional neuroimaging literature indicates that cortical midline structures (CMS) play an essential role in such self‐referential processing (Northoff & Panksepp, 2008; Northoff et al., 2006; Panksepp & Northoff, 2009). Several functional magnetic resonance imaging (fMRI) studies found that the medial prefrontal cortex (mPFC) and, specifically, the ventromedial prefrontal cortex (vmPFC) are involved in self‐referential processing, particularly if the content is emotional (Araujo, Kaplan, Damasio, & Damasio, 2015; D'Argembeau et al., 2011; Fossati et al., 2003; Kim & Johnson, 2015; van der Meer, Costafreda, Aleman, & David, 2010). Interestingly, previous fMRI studies indicate that the mPFC is also involved in emotion regulation and extinction learning (Garvert, Moutoussis, Kurth‐Nelson, Behrens, & Dolan, 2015; Ochsner, Silvers, & Buhle, 2012; Peters, Kalivas, & Quirk, 2009), two processes that are found to be dysfunctional in psychopathological states with disrupted self‐referential processing like depression or anxiety disorders (Hofmann et al., 2013).

Psychopathological states such as depression are both functionally (Grimm et al., 2009) and structurally (Qiu et al., 2014; Wagner et al., 2012) related to alterations in the CMS structures. The assessment of structural brain change such as cortical thickness has been suggested to be a promising marker for detecting the early onset of diseases (Bruce Fischl & Dale, 2000). Moreover, prior studies showed that change in brain structure could be induced through the training of specific psychological abilities, for example, mindfulness‐based attention, prosocial behavior, perspective‐taking, and multitasking performance (Valk et al., 2017; Verghese, Garner, Mattingley, & Dux, 2016). Hence, brain structure is responsive to both internal and training‐induced change. This study therefore aimed at investigating whether training‐induced change in people's self‐concept is also represented in structural brain change.

In contrast to evidence from the functional neuroimaging literature presented above, findings on the relation between gray matter structure in the CMS—including mPFC, anterior cingulate cortex (ACC), and posterior cingulate cortex (PCC)—and self‐referential processing are less consistent. Probing the structural basis of training‐induced plasticity in self‐related processes can further inform this field.

For example, higher self‐esteem was shown to be associated with increased gray matter volume in ACC, right lateral prefrontal cortex (lPFC), right hippocampus, and left hypothalamus (Agroskin, Klackl, & Jonas, 2014). In another study, higher self‐esteem was linked to greater hippocampal but smaller right amygdala volume (Wang, Kong, Huang, & Liu, 2015). In addition, the tendency to disclose information about oneself was shown to be related to increased gray matter volume in left postcentral gyrus (PCG), but negatively related to gray matter volume in right orbitofrontal cortex (OFC), which comprises the vmPFC (Wang et al., 2014). Interestingly, self‐referential processing was also associated with meta‐cognition, which is known to be related to greater gray matter volume (Fleming & Dolan, 2012; Fleming, Weil, Nagy, Dolan, & Rees, 2010) and cortical thickness (Valk, Bernhardt, Böckler, Kanske, & Singer, 2016) of the mPFC. The above studies provide preliminary evidence that different types of self‐referential processing could have different underlying structural brain correlates. However, further studies are needed to better disentangle how different aspects of self‐referential processing are related to brain structure, and how these function–structure relationships can be systematically altered through targeted training. To close this gap, we investigated how change in emotional self‐concept content elicited through socio‐cognitive training is related to structural plasticity in the brain with a particular focus on CMS.

In recent years, contemplative practices such as mindfulness‐based meditation have been increasingly studied within the domain of psychology and neuroscience (Dahl, Lutz, & Davidson, 2015; Lutz, Slagter, Dunne, & Davidson, 2008; Vago & Silbersweig, 2012). From a secular and scientific point of view, contemplative practices can be regarded as types of mental training that can alter different psychological capacities (Dahl et al., 2015; Davidson & Kaszniak, 2015; Singer et al., 2016). Currently, it is discussed that different types of contemplative practices include different components and therefore may train psychological capacities in different ways (Dahl et al., 2015; Hildebrandt, McCall, & Singer, 2017). Thus, more research is needed to disentangle which components of different contemplative practices alter which psychological capacities. Data regarding the possible influence of contemplative mental training on the emotional content of self‐referential processing are rare (Crescentini & Capurso, 2015; Dahl et al., 2015), and neuroimaging data on associated neural processes are even more limited. Previous studies have primarily focused on neural correlates of one particular type of mental training, namely mindfulness meditation (for reviews see, e.g., Fox et al., 2014; Tang, Hölzel, & Posner, 2015) and mainly applied functional neuroimaging methods. Nonetheless, the available results indicate that a decoupling between mPFC and insula may be linked to greater detachment from a narrative self‐focus after an 8‐week mindfulness‐based stress reduction training (Farb et al., 2007) and that greater activation in dorsomedial prefrontal cortex (dmPFC) could be associated with decreased reactivity toward self‐related emotions in experienced mindfulness meditators (Lutz et al., 2016). The above findings suggest that mindfulness meditation, which trains sustained attention on present‐moment awareness and a nonjudgmental stance toward one's experiences (Kabat‐Zinn, 2003) may be associated with activity in brain areas related to self‐referential processing and emotion regulation, comprising areas of the CMS. However, it is important to also consider effects of different types of mental training when investigating neuronal mechanisms underlying training‐induced behavioral change, because different types of mental training are possibly related to plasticity in different neural networks (Davidson & Kaszniak, 2015).

This study was conducted within the *ReSource Project*, a large‐scale longitudinal mental training study (Singer et al., 2016). In this study, meditation‐naïve participants underwent a 9‐month mental training which included three distinct 3‐month training modules (Singer et al., 2016). The three training modules comprised different meditation‐based core exercises which were designed to train different psychological capacities and are further described in Section 2.2. Attention and interoceptive awareness, core capacities associated with general mindfulness practices, were trained in the Presence Module. Socio‐affective capacities such as care, compassion, and gratitude, as well as prosocial motivation and dealing with difficult emotions were cultivated within the Affect Module. Finally, socio‐cognitive abilities such as meta‐cognition on one's own thoughts and perspective‐taking on self and others (also referred to as Theory of Mind or mentalizing) were trained within the Perspective Module.

In a previous study within the scope of the *ReSource project*, we assessed the influence of different core exercises taught in the three training modules on change in emotional self‐concept content assessed by extracting emotional word use from self‐descriptions using the Twenty Statement Test (Lumma, Böckler, Vrticka, & Singer, 2017). Results from this study showed a significant and specific increase in overall emotional word use after training socio‐cognitive skills in the Perspective Module in healthy adult participants (Lumma et al., 2017). During the Perspective Module, participants trained to increase meta‐cognition on their own thoughts through an “observing‐thoughts meditation” and the ability to take perspective on the self and others through a partner‐based contemplative dialogue called the Perspective Dyad (Kok & Singer, 2017; Singer et al., 2016). Specifically, the Perspective Dyad was inspired by the Internal Family System model (Schwartz, 1997) and involved participants to first identify inner aspects of the self, so‐called inner parts, and then to take the inner perspective of a given inner part to better understand how being identified with such personality aspects influences perception and interaction in daily life (Holmes, Holmes, & Eckstein, 2007). Training in both meta‐cognitive awareness of thoughts and perspective‐taking on self might have contributed to improving the participant's ability to integrate new emotional aspects into their self‐concept, allowing them to describe themselves in a more fine‐grained and differentiated emotional way (Smidt & Suvak, 2015).

The aim of this study was to assess the relationship between change in emotional self‐awareness and brain structure. Specifically, this study focused on identifying whether individual differences in behavioral plasticity of the self‐concept, observed specifically during Perspective training (Lumma et al., 2017), were predictive of training‐related change in cortical thickness in the very same participants after Perspective training.

## 2 METHODS

### 2.1 Participants

The initial sample of this study (*N* = 332) was part of the *ReSource Project*, a longitudinal mental training study conducted in the cities of Leipzig and Berlin from April 2013 to February 2016 (Singer et al., 2016). Participants were split into training and control cohorts: 80 participants were assigned to training cohort 1 (TC1), 81 participants were assigned to training cohort 2 (TC2), and an additional 81 participants were assigned to training cohort 3 (TC3). Furthermore, 90 participants were part of the retest control cohort (RCC) not undergoing any training.

The complete sample of participants underwent a screening assessment including mental health questionnaires (Major Depression Inventory (Bech, Rasmussen, Olsen, Noerholm, & Abildgaard, 2001); DIA‐X for axis I disorders for DSM‐IV (Wittchen & Pfister, 1997); and a clinical diagnostic interview (Structured Clinical Interview for DSM‐IV, SKID‐I (Wittchen, Zaudig, & Fydrich, 1997)) to ensure that they were psychologically healthy. For more details about the selection criteria, see Singer et al. (2016). All participants gave written informed consent prior to participation. The study was approved by the Research Ethics Committee of the University of Leipzig, number 376/12‐ff and the Research Ethics Committee of the Humboldt University in Berlin, numbers 2013‐02, 2013‐29, and 2014‐10. The study was registered with the Protocol Registration System of ClinicalTrials.gov under the title “Plasticity of the Compassionate Brain” with the ClinicalTrials.gov identifier: NCT01833104.

**Figure 1:**
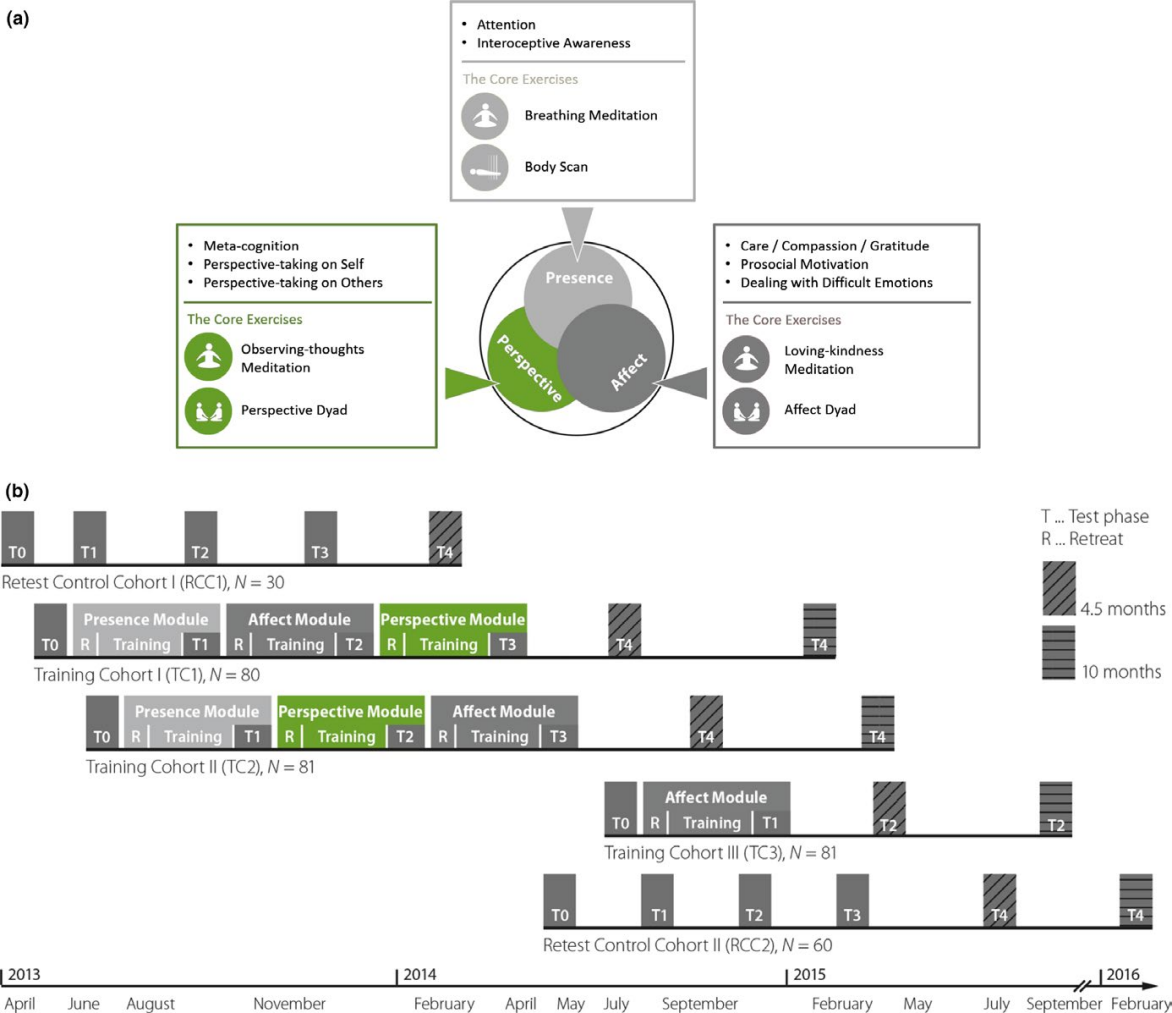
Panel (a) shows the *ReSource Project* protocol including all three training modules as well as the respective core processes and exercises. Panel (b) depicts the study design and sequence of training modules for all training cohorts (TC1, TC2, and TC3), and both retest control cohorts (RCC1 and RCC2), which were separated for organizational reasons, but combined for the current analyses. Only the Perspective Module was highlighted in green in Panel a and b, because it was of main interest within the current study. Figure courtesy of Singer et al. (2016)

The main focus of this study was to identify the underlying change in structural brain plasticity linked to training‐induced increases in self‐related emotional word use observed after training in the Perspective Module (Lumma et al., 2017). To achieve this goal, we focused only on participants who underwent training in the Perspective Module (TC1 from T2 to T3 and TC2 from T1 to T2; *N* = 161). In this study, we selected a sample that had both change scores available from the emotional word use in the TST and cortical thickness measures before and after training in the Perspective Module (for a full description of the study design, see Design & Training Protocol and Figure 1, Panel b). Of these *N* = 161 participants, *N* = 121 participants had cortical thickness change data and *N* = 136 participants had emotional word use change data after the Perspective Module. Deviations from the initial sample are due to missing data or dropout (see Chapter 4 in Singer et al., 2016) and unusable MRI or unusable behavioral data. Behavioral data were not included if responses of the TST were incomplete (for details, see the data preprocessing of the Twenty Statements Test below). MRI data were excluded if the data did not pass quality control (Bernhardt et al., 2014; Valk, Di Martino, Milham, & Bernhardt, 2015; Valk, Bernhardt, Böckler, Trautwein, et al., 2016) by two independent expert raters due to excessive movement or artefacts in the T1‐weighted MRI images (see Table 1 for details about dropout as well as missing data and available data quality). Consequently, brain–behavior correlations could be calculated in a final sample of *N* = 110 participants who had both available behavioral and brain data after training in the Perspective Module.

**Table 1:** Sample size and missing data per time point for TST and cortical thickness data

	T0	T1	T2	T3
TST—recruited				
Total (*N* = 332)	318	299	223	216
TC1 (*N* = 80)	74	68	72	68
TC2 (*N* = 81)	80	74	74	73
TC3 (*N* = 81)	78	76	—	—
RCC (*N* = 90)	86	81	77	75
TST—reasons for unavailability				
Missing	9 (5)	24 (12)	21 (16)	26 (23)
Incomplete answers	5	9	7	9
Cortical thickness—recruited				
Total (*N* = 332)	304	283	204	199
TC1 (*N* = 80)	77	69	65	59
TC2 (*N* = 81)	74	69	69	68
TC3 (*N* = 81)	72	70	—	—
RCC (*N* = 90)	81	75	70	72
Cortical thickness—reasons for unavailability				
MR incidental finding	8	8	5	5
MRI quality control	7	6	4	2
Dropout	2	12	16	23
Medical reasons	2	10	15	15
Other	9	13	7	7

The initial overall sample size at T0 was *N* = 332. Missing data for the TST were either due to participants not properly completing the TST at the respective time point or to study dropouts (D; denoted in brackets). Dropouts were due to participants developing new medical problems, discomfort with study or experiments, time constraints, or other reasons (such as moving to another city, and no disclosure). For a detailed description of the *ReSource Project* study sample, please refer to Chapter 7 in Singer et al. (2016). Cortical thickness data were excluded based on the following reasons: MR incidental findings based on T0 radiological evaluations; scans that did not survive MRI quality control because of excessive movement and/or artifacts in the T1‐weighted MRI images; dropout details can be found in (1); no MRT: due to illness/scheduling issues/discomfort in scanner; other: nondisclosed.

In a previous analysis (Lumma et al., 2017), only the Perspective Module (and thus none of the other modules) was found to induce training‐related change in emotional word use. Because only TC1 and TC2 underwent the Perspective Module (from T1 to T3), and only TC1, TC2, and RCC underwent the full length of the study, data from TC3 were not part of the present analyses. Confirmatory analyses of the behavioral results observed before (Lumma et al., 2017) were therefore only performed within participants belonging to TC1 and TC2 with available behavioral and brain change data, as well as in participants of the RCC within the same time period from T1 to T3 who again had available behavioral and brain change data.

**Figure 2:**
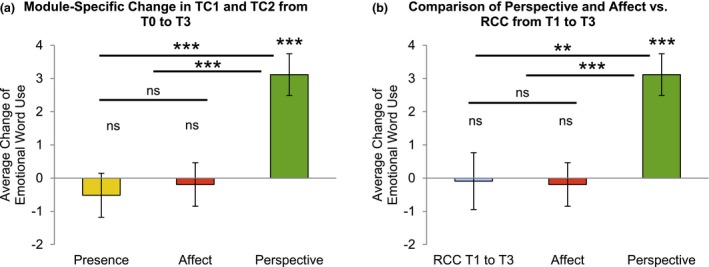
Emotional word use overall and module‐specific change in emotional word use with the revised LIWC emotion word dictionary in the reduced sample of *N* = 169 participants. Results are shown with estimated marginal means from the linear mixed model. Replicating previously published findings comprising all participants of the *ReSource Project* (Lumma et al., 2017), Panel (a) shows that change in emotional word use was again significantly greater after the Perspective Module as compared to the Presence and Affect Module and that average change in overall emotional word use was significantly different from 0 after the Perspective Module, but not after the Presence and the Affect Module. Panel (b) illustrates that change in emotional word use was greater after the Perspective Module as compared to the RCC (always calculated as cumulative change from T1–T3) and that average change in overall emotional word use was not significantly different from 0 for the RCC (***p <* *.001, **p <* *.01, *p <* *.05). ns, nonsignificant

As the present sample was reduced to those participants who had available behavioral and brain data and thus were a subsample of the previously used participant pool in Lumma et al. (2017), we first performed confirmatory behavioral analyses to assure that the observed increase in self‐related emotional word use after Perspective training but not after the other training modules and the retest control cohort (Presence, Affect, RCC) was still significant in the reduced sample (see Figure 2). For these analyses, we checked in the sample of *N* = 110 participants from TC1 and TC2 with complete Perspective change data whether they also had available Presence and Affect change data. Furthermore, we assessed which participants from the RCC had available change data from T1 to T3. The final sample for these Modules/controls was as follows. For the Presence Module (TC1 and TC2), *N* = 106 participants had available emotional word use change data, *N* = 103 had available cortical thickness change data, and *N* = 99 had both available behavioral and brain data. For the Affect Module (TC1 and TC2), *N* = 106 participants had available emotional word use change data, *N* = 104 had available cortical thickness change data, and *N* = 101 had both available behavioral and brain data. And finally, for the RCC, *N* = 72 had available emotional word use change data, *N* = 67 had available cortical thickness change data, and *N* = 59 had both available behavioral and brain data. In total, we therefore assessed data from *N* = 169 participants (94 Female; mean age 40.92 ± 9.31 years).

### 2.2 Design and training protocol

The current longitudinal mental training study, the *ReSource Project*, employed a crossover design (see Figure 1, Panel b). TC1 started with training in the Presence Module, continued with the Affect Module, and completed the training with the Perspective Module. TC2 started the training with the Presence Module, continued with the Perspective Module, and completed the training with the Affect Module. TC3 served as a replication sample and only underwent training in the Affect Module for 3 months. Further details about the design of this study can be found in Chapter 4 in Singer et al. (2016). The complete training of the *ReSource Project* lasted 9 months and was composed of three distinct 3‐month training modules (see Figure 1, Panel a). In each of the three training modules participants learned core exercises targeting either attentional and interoceptive (Presence), socio‐affective (Affect), or socio‐cognitive (Perspective) capacities. The two daily core exercises of each module were first introduced during a 3‐day retreat under the supervision of qualified meditation teachers. These core exercises should then be individually practiced, 5–6 days per week for 30 min at home. In addition, participants attended a weekly session with the meditation teachers, which allowed them to ask questions about the exercises and practice together in a group. The Presence Module was designed to train attention and interoception, and the core exercises included a breathing meditation and body scan (further details about the Presence Module can be found in chapter 3.2 in Singer et al. (2016)). The Affect Module was designed to cultivate loving‐kindness, care, gratitude, and prosocial motivation, as well as to learn how to deal with difficult emotions by practicing a loving‐kindness meditation and an Affect Dyad (Kok & Singer, 2017), which is a partner‐based contemplative dialogue (further details about the Affect Module can be found in chapter 3.3 in Singer et al. (2016)).

The main focus of this study was the effect of the Perspective Module, which will therefore be described in more detail below. The Perspective Module was designed to train the ability to take perspective of one's own inner self‐aspects and the mind of others, as well as to improve meta‐cognition about one's thought processes. The core exercises of the Perspective Module included an observing‐thoughts meditation (Krishnamurti, 1993; Ricard, 2008) and another partner‐based contemplative dialogue called the Perspective Dyad, which comprises working on the self and is based on the Internal Family System approach (Schwartz, 1997) and Inner Parts Work (Holmes et al., 2007). Essential for the Perspective Dyad is the identification of inner parts or self‐schemas that make up the personality structure as a whole. Examples of such inner parts are the “inner critic,” the “inner child,” or the “inner optimist” (Holmes et al., 2007). Initially, participants were asked to identify a set of six inner parts which they could change once per week throughout the Perspective Module. The Perspective Dyad (Böckler, Herrmann, Trautwein, Holmes, & Singer, 2017; Kok & Singer, 2017) consisted of two rounds and each participant took the role of the listener and the role of the speaker once. The speaker was asked to first briefly describe a situation that happened within the last 24 hours and was then instructed to retell the situation from the perspective of one of his/her inner parts that was randomly selected from his/her set. The listener was instructed to mindfully pay attention to the speaker and utilize perspective‐taking in order to infer which inner part was being voiced. Overall, the Perspective Dyad trained participants to flexibly enact different inner parts and take perspective on these self‐related aspects as well as improve perspective‐taking on the belief systems and thoughts of the partner. Indeed, we found that the more inner parts participants could identify in the course of the training, the better they performed in a Theory of Mind task (Böckler, Herrmann, et al., 2017).

### 2.3 Measures

#### 2.3.1 Twenty statement test

The Twenty Statement Test (TST) is an open‐ended measure that was used to assess participants’ self‐concept (Kuhn & McPartland, 1954). Participants were asked to answer the question “Who am I?” by filling in 20 statements beginning with “I am…”. Participants completed this task before and after the Perspective Module via an online‐platform specifically designed for the study (see Chapter 8 in Singer et al., 2016). Instructions were taken from Kuhn et al. (Kuhn & McPartland, 1954).

#### 2.3.2 Data pre‐processing

The twenty statements of the TST were first spell‐checked with a standard word processor. In a subsequent quality control step, statements were once more manually checked for spelling errors. Incomplete answers from the TST were not included for further analyses. Answers from the TST were regarded as incomplete if (1) participants did not provide responses to each of the 20 items, (2) words were deliberately repeated, and/or (3) nonwords were present. Emotion words of the self‐descriptions were extracted with the Linguistic Inquiry and Word Count (LIWC, Version 2015) software (Pennebaker, Booth, Boyd, & Francis, 2015). The LIWC is a widely used text analysis software counting the relative word frequency based on predefined function and content word categories (Pennebaker, Mehl, & Niederhoffer, 2003). For the extraction of emotion words in this study, we used a refined and validated (Lumma et al., 2017) version of a standard German version of the LIWC dictionary (Wolf et al., 2008). The revised LIWC emotion word dictionary has a more fine‐grained selection of words which specifically represent emotional states. We nonetheless also checked associations with the standard LIWC emotion dictionary.

### 2.4 MRI markers

#### 2.4.1 Image acquisition

Using a 3T Siemens Verio scanner (Siemens, Erlangen, Germany) with a 32‐channel head coil, we acquired a T1‐weighted 3D‐MPRAGE sequence (176 sagittal slices, repetition time [TR] = 2,300 ms, echo time [TE]=2.98 ms, inversion time [TI] = 900 ms, flip angle=7**°**, field of view [FOV] = 240 × 256 mm^2^, matrix = 240 × 256, 1 × 1 × 1 mm^3^ voxels). Throughout the duration of our longitudinal study, imaging hardware and console software (Syngo B17) were held constant.

#### 2.4.2 Cortical thickness measurements

Each T1‐weighted MRI was processed using FreeSurfer to generate cortical surface models and to measure cortical thickness. All processing was carried out on the same 32‐core computer with the same software version (5.1.0; http://surfer.nmr.mgh.harvard.edu). FreeSurfer has been validated against histological analysis (Rosas et al., 2002) and manual measurements (Kuperberg et al., 2003). Processing steps have been detailed elsewhere (Dale, Fischl, & Sereno, 1999; Fischl, Sereno, & Dale, 1999; Han et al., 2006). Following surface extraction, sulcal and gyral features of an individual were warped to an average spherical representation, fsaverage5, allowing for the accurate matching of thickness measurements across participants. Surfaces were visually inspected, and inaccuracies manually corrected. Thickness data were smoothed on tessellated surfaces using a 20 mm full‐width‐at‐half‐maximum (FWHM) Gaussian kernel, which reduces measurement noise while preserving the capacity for anatomical localization, as it respects cortical topological features (Lerch & Evans, 2005).

#### 2.4.3 Statistical analyses

Statistical analyses were performed using SurfStat for Matlab (Worsley et al., 2009). As in previous studies (Bernhardt, Bernasconi, Concha, & Bernasconi, 2010; Bernhardt, Kim, & Bernasconi, 2013), longitudinal analysis was carried out using linear mixed‐effects models, a statistical technique that allows for the inclusion of multiple measurements per subject and irregular intervals between measurements (Pinheiro & Bates, 2000). In all models, we controlled for baseline age and sex, given their established effects on brain structure (Salat et al., 2004). Details on structural change as a result of training as such are described elsewhere (Valk et al., 2017).

1. *Behavioral modulation of brain change*. To map the relationship between individual differences in behavior and change in brain structure, we first generated subject‐specific cortical thickness change maps (ΔCT) by subtracting vertex‐wise thickness maps of two given time points for a given subject, while normalizing cortical thickness scores by controlling for mean thickness at each time‐point. A similar approach was chosen to assess behavioral change *(*∆*B*). We then assessed whole‐brain modulation of ∆*CT* by ∆*B* in a given cohort:
  $$\Delta \text{CTi}=\beta_{0}+\beta_{1}\;*\text{Age}+\beta_{2}\;\;*\text{Sex}+\beta_{3}\;\;*\Delta B+\text{random(Sub)}+I$$
  To account for random effects, the above equation comprises a random effect term of subject (Sub), as well as the intercept (I).
2. *Correction for multiple comparisons*. Statistical results were corrected for multiple comparisons by means of random field theory using both a typically used (Andrews et al., 2017; Bernhardt et al., 2013; Ecker et al., 2013; Hong, Bernhardt, Schrader, Bernasconi, & Bernasconi, 2016) cluster‐determining threshold (CDT) of *p* < .025 and FWE of *p* < .05 (two‐tailed) for 20 mm FWHM smoothed surface‐based 2D thickness data where higher smoothing kernels relate to more readily fulfilled assumptions of Gaussian Random Field Theory (Eklund, Nichols, & Knutsson, 2016; Flandin & Friston, 2016; Greve & Fischl, 2017), as well as a more conservative cluster‐determining threshold recently recommended for the analysis of surface‐based anatomical cortical thickness data (Greve & Fischl, 2017) of CDT *p* < .01 and FWE *p* < .02 (two‐tailed). Moreover, we verified consistency of results across the two separate training cohorts.
3. *Effect size*. Effect sizes of whole‐brain cortical thickness change analyses were derived from *t*‐values and degrees of freedom (*df*) by means of effect size correlations *r* with the formula *r = *√(*t* ^2^/*t* ^2^ *+ df*) (Rosnow, Rosenthal, & Rubin, 2000).

#### 2.4.4 Behavioral × brain associations

Change scores of emotional word use (after minus before the Perspective Module) were calculated (see Lumma et al., 2017) and used as behavioral predictors in whole‐brain analyses in order to identify associations between change in cortical thickness and change in emotional word use. All whole‐brain analyses were calculated by controlling for sex, age, and random effects. Furthermore, all behavioral variables of interest were first checked for outliers by defining outliers as values higher or lower than three standard deviations from the mean. Any raw data values that were labeled as outliers using this outlier labeling rule were then winsorized to the respective three standard deviation upper and/or lower boundaries.

For the confirmatory analyses of the previously published behavioral results focusing on average module‐specific training‐related change in emotional self‐related word use (Lumma et al., 2017), we used linear mixed models (including the intercept) with the fixed factor Module (Perspective, Presence, Affect, and the RCC), and sex as well as mean‐centered age as control variables of no interest. Linear mixed models (LMMs) were run in SPSS (IBM Corp, 2013) and post hoc contrasts within the LMMs were not corrected for multiple comparisons. Within the multilevel model framework used here, estimates are “shrunk” toward a common mean; this “partial pooling” corrects for the increased risk of false positives typically incurred by multiple comparisons without compromising power (Gelman, Hill, & Yajima, 2012). Separate one‐sample *t*‐tests were used to determine whether the average change per module significantly differed from 0. The one‐sample *t*‐tests were Bonferroni corrected. Effect sizes of the main and interaction effects were calculated with Omega‐squared (ω²) by taking the difference from 1 of the variance of the residuals of the full model divided by the variance of the residuals of the model without the respective factor of interest (Xu, 2003). A small effect size is represented by ω² ≥ .010, a medium effect size by ω² ≥ .059, and a large effect size by ω² ≥ .138 (Kirk, 1996).

## 3 RESULTS

### 3.1 Training‐related change in emotional word use with the revised LIWC emotion word dictionary

In a previous article (Lumma et al., 2017), we found that overall emotional word use only significantly increased after training in the Perspective Module. Because in this study, we had to use a reduced sample for behavior–brain correlations (see Section 2), we confirmed the previously observed specificity of overall emotional word use change after the Perspective Module with an analysis relying upon module change scores within the *N* = 169 participants included in the whole‐brain analyses (from TC1 and TC2, with Perspective change *N* = 110—including *N* = 99 with Presence and *N* = 101 with Affect change—and RCC with overall change from T1 to T3 *N* = 59—see Section 2). All above analyses were conducted by relying upon a refined and validated LIWC emotion word dictionary (Lumma et al., 2017). For completeness, we recalculated all analyses with the standard LIWC emotion word dictionary. The detailed results of all these calculations using the standard LIWC can be found in the Appendix S1.

The LMM with the fixed factor Module (Perspective, Presence, Affect, and RCC) revealed a significant main effect of Module (*F*(3, 363) = 6.98, *p *<* *.001) with a small effect size (ω^2^ = 0.055). Post hoc contrasts were specified in order to compare the Perspective Module‐specific change in TC1 and TC2 to change in the other training modules and in the RCC. Change in emotional word use after the Perspective training in TC1 and TC2 was significantly greater as compared to the RCC (*t*(363) = 3.02, *p *=* *.003, 95% CI [1.118, 5.291]) and as compared to the Presence training (*t*(363) = 3.99, *p *<* *.001, 95% CI [1.840, 5.420]) as well as greater as compared to the Affect training (*t*(363) = 3.65, *p *<* *.001, 95% CI [1.525, 5.086]). Change in emotional word use after the Affect training in TC1 and TC2 was not significantly greater as compared to the RCC (*t*(363) = −0.094, *p *=* *.925, 95% CI [−2.219, 2.017]), and not significantly different from the Presence training (*t*(363) = 0.35, *p *=* *.727, 95% CI [−1.503, 2.152]). Additional post hoc one‐sample *t*‐tests were run for each module and the RCC to determine whether the average changes scores of emotional word use were significantly different from 0. Results showed that overall emotional word use was significantly different from 0 after the Perspective Module (*t*(109) = 4.212, *p *<* *.001, 95 CI [1.649, 4.579]), but not after the Presence Module (*t*(98) = −.808, *p *= n.s., 95% CI [−1.786, .753]) and the Affect Module (*t*(100) = −.290, *p *= n.s., 95% CI [−1.487, 1.107]). In addition, average change scores of overall emotional word use were not significantly different from 0 in the RCC (*t*(58) = −.177, *p *= n.s., 95% CI [−1.073, 0.899]). *p*‐Values for these additional post‐hoc one‐sample *t*‐tests were Bonferroni‐corrected. Results are depicted in Figure 2, Panels a and b. In sum, we could replicate previous findings of Perspective Module‐specific increases in emotional self‐related word use in the TST in the present reduced sample.

### 3.2 Behavioral × brain associations following perspective training

#### 3.2.1 Whole‐brain association between overall emotional word use change and cortical thickness change

**Figure 3:**
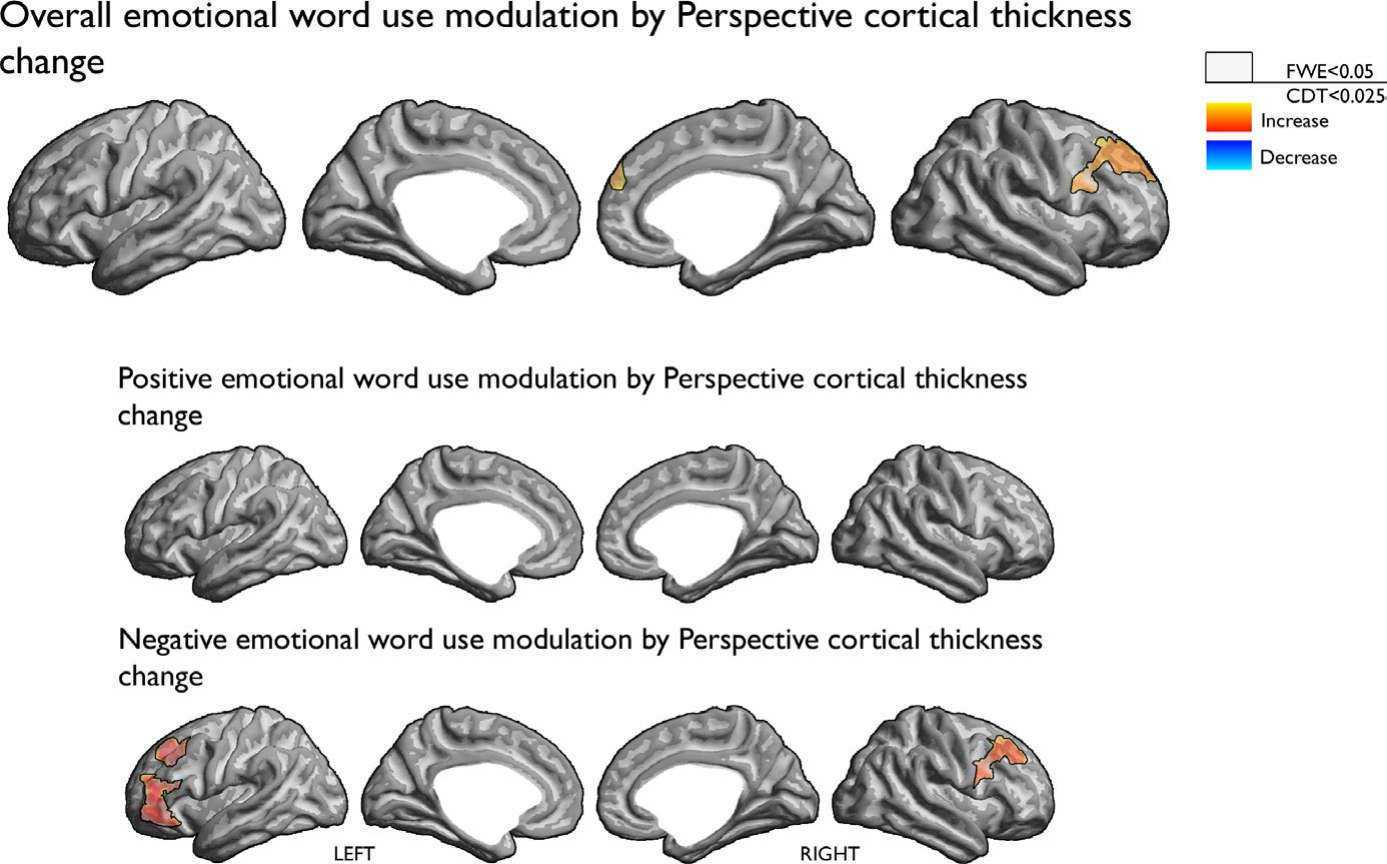
Relationship between cortical thickness increase and increase in (a) overall emotional word use (top), positive emotional word use (middle), and negative emotional word use (bottom) after training in the Perspective Module. To correct for multiple comparisons, uncorrected findings at the cluster level (cluster‐determining threshold [CDT] of *p* < .025) were additionally thresholded at the cluster level with FWE *p* < .05 (two‐tailed) using random field theory for nonisotropic images (black outlines)

Investigating the association between cortical thickness change and overall emotional word use change after the Perspective training on the whole‐brain level revealed a significant cluster in right medial prefrontal cortex (mPFC) extending into dorsolateral PFC (dlPFC) (see Table 2 and Figure 3). The relationship between change in overall emotional word use and change in cortical thickness in the right mPFC‐dlPFC cluster is consistent across training cohorts (see Appendix S3).

**Table 2:** Brain areas with significant correlations between cortical thickness change and emotional word use change after Perspective training (corrected for multiple comparisons)

Word usage	Region	*x*	*y*	*z*	*t*‐Value	Surface area (mm^2^)	Effect size
Overall emotional words	*R superior frontal gyrus (peak)* R middle frontal gyrus, R inferior frontal gyrus (pars opercularis), R inferior frontal gyrus (pars triangularis), R medial frontal gyrus	22	33	36	3.51	1997	*df* = 106, *r* = .32
Negative emotional words	*L inferior frontal gyrus, pars orbitalis (peak)* L superior frontal gyrus, L middle frontal gyrus, L middle frontal gyrus (orbital part), L inferior frontal gyrus (pars triangularis)	−42	42	−8	3.06	2880	*df* = 106, *r* = .28
Negative emotional words	*R middle frontal gyrus (peak)* R superior frontal gyrus, R inferior frontal gyrus (pars opercularis), R inferior frontal gyrus (pars triangularis), R rolandic operculum	22	31	38	4.41	2040	*df* = 106, *r* = .39

Overview of brain regions showing structural change associated with overall and negative emotional word use after Perspective training. Information including the coordinates, *t*‐values, surface area, and effect sizes is provided for the peaks of the regions, which were identified with the automated anatomical labeling (AAL) database. *df*, degrees of freedom.

#### 3.2.2 Whole‐brain association between positive and negative emotional word use change and cortical thickness change

As an additional analysis, we then investigated the relationship between cortical thickness change and positive and negative emotional word use change after the Perspective Module on the whole‐brain level. This analysis revealed that negative emotional word use change was significantly correlated with cortical thickness change in the right dlPFC and the left pars orbitalis, which extended into the left dlPFC after Perspective training (see Table 2 and Figure 3). The relationship between change in negative emotional word use and change in cortical thickness in the right dlPFC and the left pars orbitalis clusters are consistent across training cohorts (see Appendix S3). The whole‐brain analysis regarding positive emotional word use change did not reveal any significant clusters (Figure 3). An additional whole‐brain analysis was run to test for a valence‐specific effect using valence difference scores (positive emotional word use vs. negative emotional word use), and the results did not show any significant clusters.

## 4 DISCUSSION

In the current study, we investigated structural plasticity underlying training‐induced change in the emotional self‐concept after daily perspective‐taking practice over 3 months. Specifically, the study was based on observed increases in emotional word use in the Twenty Statement Test (TST; Kuhn & McPartland, 1954) after a 3‐month socio‐cognitive mental training module (Perspective Module) in a large sample (Lumma et al., 2017) that was part of a 9‐month longitudinal mental training study, the *ReSource Project* (Singer et al., 2016), including three distinct 3‐month mental training modules (Presence, Affect, and Perspective). Extending these previous findings, present analyses of training‐related cortical thickness change revealed that such change in emotional self‐concept content was associated with increased gray matter in right medial prefrontal cortex (mPFC) extending into right dorsolateral PFC (dlPFC) after the Perspective Module. Moreover, we found a positive relationship between change in negative emotional word use and cortical thickness change in the right dlPFC and the left pars orbitalis extending into the left dlPFC after the Perspective Module. Even though significant effects were only found for negative emotional words and not for positive emotional words when conducting independent analyses for each valence, a subsequent analysis on valence difference scores did not reveal any significant effects.

The core mental exercises of the Perspective Module were designed to train socio‐cognitive skills and included an observing‐thoughts meditation and a partner‐based contemplative exercise called the Perspective Dyad. The observing‐thoughts meditation aimed at training meta‐awareness about one's thought processes as well as the ability to detach from one's thoughts (Ricard, 2008). The Perspective Dyad was developed to train perspective‐taking onto aspects of the self and the mind of others and was inspired by both work in the therapeutic domain such as the Internal Family Systems model (IFS) (Holmes et al., 2007; Schwartz, 1997) and research about socio‐cognitive abilities such as perspective‐taking on the beliefs, thoughts, and intentions of others (Frith & Frith, 2003; Singer, 2012). Overall, the Perspective Module can be regarded as an intervention that selectively trains perspective‐taking on different aspects of the self as well as on the mind of others (see also Böckler, Herrmann, et al., 2017; Kok & Singer, 2017; Singer et al., 2016).

The results from the current study show that this training indeed induced plasticity of the self‐concept in brain and behavior. Change in overall emotional word use was associated with increased cortical thickness in the right mPFC extending into right dlPFC. Previous fMRI studies have related the mPFC to various processes involved in self‐ and other‐related cognition. For example, the mPFC was implicated in self‐referential processing (van der Meer et al., 2010; Northoff et al., 2006; Qin & Northoff, 2011) and attending to emotional responses related to the self (Satpute, Shu, Weber, Roy, & Ochsner, 2013) and emotion regulation (Ochsner et al., 2012). Furthermore, the mPFC is known to be functionally (Denny, Kober, Wager, & Ochsner, 2012; Kanske, Böckler, Trautwein, & Singer, 2015; Mitchell, Macrae, & Banaji, 2006; Yaoi, Osaka, & Osaka, 2009) and structurally (Valk, Bernhardt, Böckler, Trautwein, et al., 2016) involved in socio‐cognitive abilities, as well as meta‐cognition on perceptual information (Fleming & Dolan, 2012; Valk, Bernhardt, Böckler, Kanske, et al., 2016). Interestingly, supporting the close link between self‐ and other‐referential processing that is related to increased mPFC activity, Böckler and colleagues observed that an increased understanding of one's inner parts (e.g., increased amount of identified inner parts) during the 3‐month Perspective Module was associated with increased perspective‐taking on others in a laboratory‐based Theory of Mind task (Böckler, Herrmann, et al., 2017).

The association between change in overall emotional word use and change in right mPFC gray matter also extended into adjacent dlPFC. Interestingly, the right dlPFC was more strongly associated with increased negative emotional word use after the Perspective Module and is known to be related to self‐referential processing (Schmitz, Kawahara‐Baccus, & Johnson, 2004; Wu et al., 2015) as well as to self‐control (Steinbeis, Bernhardt & Singer, 2012) and emotion regulation (Moore et al., 2016; Ochsner & Gross, 2005; Ochsner et al., 2012). In a previous study, dlPFC and also mPFC activity were related to cognitive reappraisal and mindfulness as strategies to regulate one's emotions (Opialla et al., 2015). Furthermore, prior research has shown that the dlPFC indirectly modulates amygdala activity through connections with the vmPFC (Buhle et al., 2014). Engaging the dlPFC might be particularly important to exert cognitive control in terms of cognitive reappraisal of negative emotional self‐content throughout the practice of the daily Perspective Dyad (Böckler, Herrmann, et al., 2017; Holmes et al., 2007). More specifically, the dlPFC might support the ability to inhibit the automatic and unconscious classification of mental content as either positive or negative, which could allow for the conscious and more volitional reappraisal of emotional content, thereby helping it to become better integrated into the participant's self‐concept (Moore et al., 2016; Ochsner & Gross, 2005; Ochsner et al., 2012).

Besides the above effects emerging from the analysis with overall emotional word use change, the analysis using negative emotional word use change revealed an additional positive association between change in self‐descriptions and change in cortical thickness in the left pars orbitalis extending into the left dlPFC and the right dlPFC—a pattern that was not present when separately testing for positive emotional word use. The pars orbitalis is an area known to be related to semantic and emotional processing by acting as a point of convergence between different networks for processing semantic and emotional content (Belyk, Brown, Lim, & Kotz, 2017). Such mechanisms, together with cognitive control are likely sustained by the dlPFC (see above), appear to have been more strongly recruited for negative as compared to positive emotional self‐descriptions after training in the Perspective Module. However, a direct comparison between negative and positive emotional word use on the whole‐brain level was not significant, which suggests that the left pars orbitalis and bilateral dlPFC also sustain functions related to positive emotional self‐referential processing—albeit possibly to a somewhat lesser degree than for negative information. A more general role of the dlPFC during emotion regulation could also be supported by previous evidence, which showed that the dlPFC is involved during reappraisal of both negative and neutral stimuli (Golkar et al., 2012). Future studies are needed to more precisely delineate associations between training‐induced change in self‐descriptions and structural brain change as a function of emotional valence.

Based on the seminal publication by Eklund et al. (2016) regarding inflated false‐positive rates in functional magnetic resonance imaging (fMRI) research, a recent paper directly evaluated false‐positive rates in surface‐based anatomical analysis comprising cortical thickness data (Greve & Fischl, 2017). In the latter publication, the authors note that “for thickness, the [false‐positive rates] FPRs were in the range of 10% instead of the desired 5% for typical analysis parameters, much less than has been found for fMRI or VBM analysis at matching cluster forming thresholds [CFTs]” (p. 21). In line with the above, the data described in our manuscript using a CFT/CDT of *p* < .025 with *p* < .05 for family‐wise‐error (FWE) correction (two‐tailed) should have resulted in false‐positive rates no larger than 5%–10%. The applied thresholds furthermore conform to previous research on other (Andrews et al., 2017; Bernhardt et al., 2013; Ecker et al., 2013; Hong et al., 2016) as well as the same data set (Valk et al., 2017), particularly for 20 mm FWHM smoothed surface‐based 2D thickness data where higher smoothing kernels relate to more readily fulfilled assumptions of Gaussian Random Field Theory (Eklund et al., 2016; Flandin & Friston, 2016; Greve & Fischl, 2017). In addition, the effects observed at the above thresholds within the three brain areas of interest are significant for each training cohort separately as evident from post hoc analyses—except for one association at a marginal level of *p* = .054. These results show that the overall patterns persist in each training cohort independently, which provides an additional data validity check. We therefore believe that the reported results at p‐CFT/CDT < .025 and p‐FWE < .05 (two‐tailed) are solid. Nonetheless, based on the recommendations by Greve and Fischl (2017), especially that “… for a thickness study with CFT ≤ .01 and FWMH ≥ 6, a cluster would need to have a nominal *p*‐value of .02 or less to be truly significant at the .05 level” (p. 13), we reran the cortical thickness analyses with p‐CFT/CDT<. 01 and p‐FWE< .02 (two‐tailed) (see Appendix S4). For this additional analysis with a more stringent p‐CFT/CDT and p‐FWE, only one cluster in the right middle frontal gyrus/dlPFC related to negative emotional word use remained significant. These findings indicate that the right dlPFC associated with negative emotional word use was the most reliable cluster in the present study, possibly also driving the association between cortical thickness change and the change in overall word use within the same cluster as reported above. Nonetheless, as mentioned by Greve and Fischl (2017), “the problem with such stringent CFTs is that there are often no voxels that survive; this is a huge disadvantage as it will greatly reduce [the true positive rate] TPR/power” (p. 19). The application of a more stringent CFT/CDT in this study may thus have removed some valid positive results. Generally speaking, findings from this study should be regarded as an initial exploratory contribution to the question how change in emotional self‐concept content is related to change in brain structure until further evidence can be provided, also by means of future meta‐analyses of similar results.

Another crucial question for future research is whether the identified changes in emotional self‐concept content and the associated changes in brain structure serve a psychologically adaptive function. As described in the introduction, psychopathological conditions such as depression are related to altered self‐referential processing (Nejad et al., 2013) and alterations in the CMS structures (Grimm et al., 2009; Qiu et al., 2014). Specifically, depression is characterized by a heightened focus on the self and a strong identification with negative thoughts and beliefs (Beck, 2008). It could be the case that meditation training initially induces a heightened self‐focus (Vago & Silbersweig, 2012), which subsequently declines with practice over time as shown in expert meditators (Farb et al., 2007). Future studies should investigate whether the observed behavioral and brain changes are adaptive, for instance, in that they reduce affective and cognitive patterns related to depression, and whether the socio‐cognitive exercises of the Perspective Module are suited for clinic populations as well.

In the present investigation, we used a refined and previously validated (Lumma et al., 2017) LIWC emotion word dictionary. This approach was chosen because some of the emotion words from the standard LIWC emotion word dictionary include words that reflect emotionally valenced personality aspects such as “honest,” “wise,” or “fanatic,” which represent emotional personality characteristics. However, we were specifically interested in emotional feeling states and therefore carried out a more fine‐grained selection of emotion words. Thus, the revised LIWC emotion word dictionary only includes emotion words reflecting emotional feeling states such as “joyful,” “sad,” and “pleasant” and was successfully validated by two independent raters for overall emotional, positive emotional, and negative emotional words (Lumma et al., 2017). Using this revised dictionary, we could replicate our previous behavioral findings pertaining to a specific Perspective effect on overall emotional word use in the reduced participant sample on *N* = 169 (see Section 3 and Appendix S1.1). Nonetheless, we also performed the same behavioral analyses with the standard LIWC emotion word dictionary, which revealed the same pattern of results (see Appendix S1.2). Whole‐brain analyses on brain × behavior associations were also recalculated using the standard LIWC emotion word dictionary (see Appendix S2). The same trends toward a relationship between change in emotional word use and cortical thickness change as for the revised emotion words could be observed, but did not survive whole‐brain correction for multiple comparisons. Furthermore, when specifically testing for associations between emotional word use change using the standard LIWC dictionary and cortical thickness change in the Perspective effect areas derived with the revised LIWC emotion word dictionary, there were significant associations in the right dlPFC and left pars orbitalis pertaining to negative emotional word use change, and a marginally significant association in the right mPFC‐dlPFC pertaining to overall emotional word use change. These additional analyses generally confirm the validity of the revised LIWC emotional word dictionary. The fact that the revised LIWC emotional word dictionary showed stronger effects in the present investigation likely was due to its refined properties, allowing it to more specifically pick up emotional states that may better characterize changes in the emotional evaluation of one's self‐concept (as compared to more general emotion words used in the standard LIWC emotion word dictionary).

In addition, it should be pointed out that several other brain areas are probably also involved in the above described self‐referential, emotional, and cognitive processes, as these processes are likely to interact on several levels of neural computation (Lindquist, Wager, Kober, Bliss‐Moreau, & Barrett, 2012). Thus, in contrast to these structural brain analyses, future research on training‐related plasticity in self‐concept should involve additional functional neuroimaging methods and also focus on the overlap between the coarseness of subjective measures with the coarseness of neuronal and physiological measures (Bitbol & Petitmengin, 2017). For example, personality questionnaires measure traits and vary in different features from other subjective measures such as state questionnaires, which measure momentary psychological states, or in‐depth qualitative interviews (Petitmengin, 2006), which capture more fine‐grained aspects of subjective experiences. Neural and physiological measures also differ with respect to the features they can assess, including, for instance, the temporal domain. Differences in features of subjective, neuronal, and physiological measures should be considered when trying to establish a relationship between these measures. The use of resting‐state fMRI and connectivity analyses might for instance better match the degree of coarseness of the emotional word use measure and reveal additional information about training‐induced restructuring of brain networks.

To conclude, findings from our study embedded in the large‐scale 9‐month *ReSource Project* (Singer et al., 2016) show that a 3‐month socio‐cognitive mental training intervention that focuses on increasing the ability to take a meta‐cognitive perspective on aspects of the self and others, can induce change in emotional self‐descriptions and induce concomitant structural brain change by increasing cortical thickness in regions such as the mPFC, dlPFC, and pars orbitalis known to be involved in self‐referential processing, emotion regulation, and cognitive control. This evidence for behavioral and structural brain plasticity in the domain of the self‐concept after mental training can guide future development of behavioral interventions in therapeutic settings and the domain of self‐concept.
