# Supplementary material for: Change in emotional self‐concept following socio‐cognitive training relates to structural plasticity of the prefrontal cortex
Source: Brain Behav. 2018 Mar 13;8(4):e00940. doi: 10.1002/brb3.940 (PMC5893336; doi:10.1002/brb3.940)
Supplement: Supplementary file 1 [file BRB3-8-e00940-s001.doc]

Supplementary Material

**Change in Emotional Self-Concept**

**following Socio-Cognitive Training relates to**

**Structural Plasticity of the Prefrontal Cortex**

Anna-Lena Lumma^a,b *^, Sofie L. Valk^a*^, Anne Böckler^a,c^, Pascal Vrtička^a^, &

Tania Singer^a^

^a^Department of Social Neuroscience, Max Planck Institute for Human Cognitive and Brain Sciences, Stephanstraße 1a, PO BOX 50 03 55, 04303 Leipzig, Germany

^b^University of Witten/Herdecke, Department of Psychology and Psychotherapy,

Alfred-Herrhausen-Straße 50, 58452 Witten, Germany

^c^University of Würzburg, Department of Psychology III, Röntgenring 11,

97070 Würzburg, Germany

* Anna-Lena Lumma and Sofie L. Valk contributed equally to this

work and agreed on a shared first-authorship.

**Supplementary information contains:**

S1. Confirmatory Results of Change in Emotional Word Use

S2. Relationship between Cortical Thickness Change and Emotional Word Use Change using the Standard LIWC Emotion Word Dictionary

S3. Robustness of Relationship between Cortical Thickness Change and Emotional Word Use Change

S4. Confirmatory Analysis of the Relationship between Cortical Thickness Change and Emotional Word Use Change

**S1. Confirmatory Results of Change in Emotional Word Use**

In the main results, we report training-induced increase in overall emotional word use after the Perspective Module in a reduced sample of N= 169 participants (see Results section). These findings confirm results published in a previous article relying on a larger sample of participants of the *ReSource* *Project* (Lumma et al., 2017). Here, we show the results of additional analyses including: S1.1) change in valence difference scores of emotional word use with the revised LIWC emotion word dictionary; S1.2.1) training-related change in emotional word use with the standard LIWC emotion word dictionary; and S.1.2.2) change in valence difference scores of emotional word use with the standard LIWC emotion word dictionary.

**S1.1. Training-related Change in Valence Difference Scores of Emotional Word Use with the Revised LIWC Emotion Word Dictionary**

Because we found a significant effect of Module for overall emotional word use (driven by specifically increased emotional word use after Perspective training; see main Results section), we conducted an additional exploratory analysis to check for valence-specific differences by using valence difference scores (positive emotional word use vs. negative emotional word use). Positive valence difference scores indicate greater positive emotional word use in contrast to negative emotional word use and negative valence difference scores indicate greater negative emotional word use in contrast to positive emotional word use. A linear mixed model with the fixed factor Module (Perspective, Presence, Affect, and RCC) showed a significant main effect of Module (*F*(3, 363) = 3.50, *p* = .016) with a small effect-size (ω² = 0.028). The post-hoc contrasts revealed that change in valence difference scores after the Perspective training in TC1 and TC2 was significantly greater as compared to the Presence training (*t*(363) = 3.23, *p* = .001, 95% CI [1.046, 4.294]), but not significantly greater as compared to the Affect training (*t*(363) = 1.59, *p* = .113, 95% CI [-0.309, 2.922]) and not significantly greater compared to the RCC (*t*(363) = 1.51, *p* = .133, 95% CI [-0.442, 3.344]). In addition, change in valence difference scores after the Affect training in TC1 and TC2 was not significantly greater as compared to the RCC (*t*(363) = 0.148, *p* = .882, 95% CI [-1.777, 2.066]), and not significantly different from the Presence training (*t*(363) = 1.62, *p* = .107, 95% CI [-0.294, 3.022]). Results showed a marginal significant difference from 0 in overall emotional word use after training in the Perspective Module *t*(109) = 2.485, *p* = .056, 95% CI [.336, 2.987], but not after training in the Presence Module *t*(98) = -1.809, *p* = .296, 95% CI [-2.108, .098] and in the Affect Module *t*(100) = .594, *p* = n.s. , 95% CI [-.860, 1.595]. Average change scores of overall emotional word use were also not significantly different from 0 for the RCC *t*(58) = .550, *p* = n.s. , 95% CI [-.6658, 1.1704]. P-values for the one-sample t-tests were Bonferroni-corrected.

Overall, these findings indicate a valence-specific effect with greater positive vs. negative emotional word use after training in the Perspective Module compared to training in the Presence Module in a sample of N = 169 participants used in the present study. In a previous publication using the entire sample of the *ReSource* *Project*, no valence-specific effect after training in the Perspective Module was observed (Lumma et al., 2017).

**S 1.2.1 Training-related Change in Emotional Word Use with the Standard LIWC Emotion Word Dictionary**

The confirmation of the previously observed specificity of overall emotion word use change after the Perspective Module was also repeated using the standard LIWC emotion word dictionary in the sample of N = 169.

The linear mixed model with the fixed factor Module (Perspective, Presence, Affect, and RCC) revealed a significant main effect of Module (*F*(3, 363) = 2.963, *p* = .032) with a small effect-size (ω² = 0.024). The same post-hoc contrasts used above were specified in order to compare the Perspective Module-specific change in TC1 and TC2 to change in the other training modules and in the RCC. Change in emotional word use after the Perspective training in TC1 and TC2 was significantly greater as compared to the RCC (*t*(363) = 2.16, *p* = .032, 95% CI [0.301, 6.596]), as well as compared to the Presence training (*t*(363) = 2.51, *p* = .012, 95% CI [0.749, 6.149]) and as compared to the Affect training (*t*(363) = 2.35, *p* = .019, 95% CI [0.527, 5.900]). Change in emotional word use after the Affect training in TC1 and TC2 was not significantly greater as compared to the RCC (*t*(363) = 0.14, *p* = .885, 95% CI [-2.960, 3.430]), and not significantly different from the Presence training (*t*(363) = 0.17, *p* = .867, 95% CI [-2.522, 2.992]). Post-hoc one-sample t-tests were run for each module and the RCC to determine whether emotional word use was significantly different from 0. Results showed that overall emotional word use was significantly different from 0 after training in the Perspective Module (*t*(109) = 3.153, *p* = .008, 95 CI [1.230, 5.393]), but not after training in the Presence Module (*t*(98) = -.130, *p* = n.s., 95 CI [-2.159, 1.894]) and in the Affect Module (*t*(100) = .096, *p* = n.s., 95 CI [-1.914, 2.109]). In addition, average change scores of overall emotional word use were not significantly different from 0 for the RCC (*t*(58) = -.160, *p* = n.s., 95 CI [-1.639, 1.396]). P-values for the one-sample t-tests were Bonferroni-corrected. Results are depicted in Figure S1.2.1, Panels A and B. The findings with the sample of N = 169 confirm the previously observed specificity of overall emotion word change after the Perspective Module using the entire sample of the *ReSource Project* (Lumma et al., 2017).


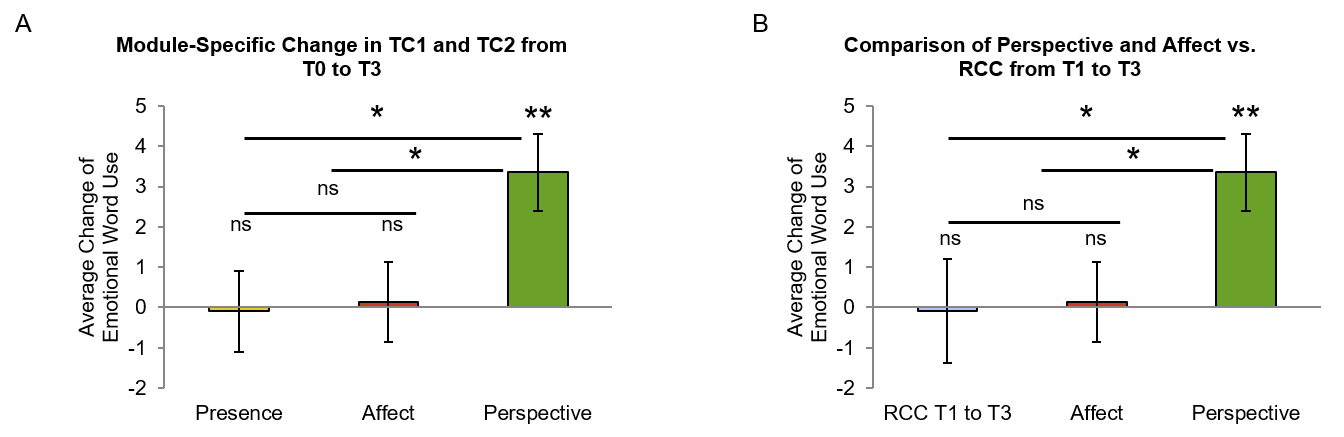


*Figure S1.2.1.* Emotional word use overall and module-specific change in emotional word use with the standard LIWC emotion word dictionary. Results are shown with estimated marginal means from the linear mixed model. Panel A shows that change in overall emotional word use was greater after the Perspective Module as compared to the Presence Module and the RCC and that average change in overall emotional word use was significantly different from 0 after the Perspective Module, but not after the Presence and Affect Module. Panel B illustrates that change in overall emotional word use was greater after the Perspective Module as compared to the RCC (always calculated as cumulative change from T1-T3) and that average change in overall emotional word use was not significantly different from 0 for the RCC. (****p* < .001, ***p* < .01, **p* < .05, ns= nonsignificant).

**S 1.2.2 Training-related Change in Valence Difference Scores of Emotional Word Use with the Standard LIWC Emotion Word Dictionary**

The additional exploratory analysis to check for valence-specific differences by using valence difference scores (positive emotional word use vs. negative emotional word use) was rerun with emotional word use scores extracted with the standard LIWC emotion word dictionary. A linear mixed model with the fixed factor Module (Perspective, Presence, Affect, and RCC) showed no significant main effect of Module (*F*(3, 363) = 1.912, *p* = .127). A valence-specific effect after training in the Perspective Module was not observed in the sample of N = 169 participants used in the present study. In a previous study using the entire sample of the *ReSource* *Project* (Lumma et al., 2017) we found an increase in positive vs. negative emotion words for TC2 after training in the Perspective Module. The lack of a significant increase in positive vs. negative emotion words in the current study was likely due to the reduced N in the present sample, as a slight trend towards a module effect is also visible in the current analysis.

**S2. Relationship between Cortical Thickness Change and Emotional Word Use Change using the Standard LIWC Emotion Word Dictionary**

Pertaining to the whole-brain analyses of emotional word use change and cortical thickness change after the Perspective Module, the same steps as mentioned in the results section were repeated with the standard LIWC emotion word dictionary. Results revealed no significant clusters after correction for multiple comparisons with FWE p< .05 at the cluster-level for the associations between cortical thickness change and overall, positive, negative emotional and positive emotional vs. negative emotional word use change after the Perspective training. However, at an uncorrected level with p< .025 at the vertex, the results showed brain areas overlapping with the clusters identified using the revised LIWC emotion word dictionary for both overall and negative emotional word use (see Figure S2).


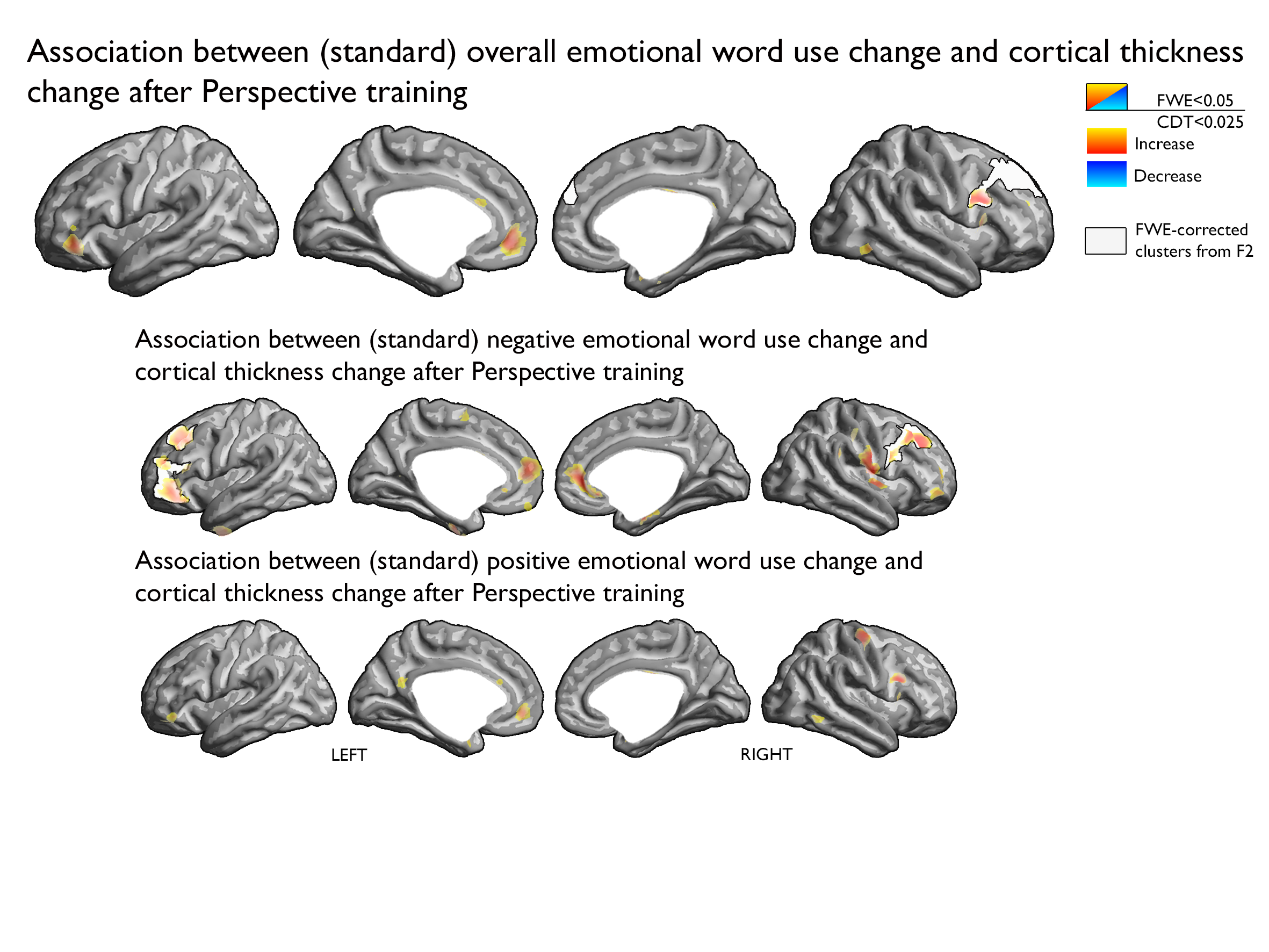


*Figure S2.* Relationship between cortical thickness increase and increase in overall emotional word use (top), negative emotional word use (middle), and positive emotional word use (bottom) after training in the Perspective Module. Trends with analyses using the standard LIWC emotion word dictionary at *p*< .025 uncorrected (cluster-determining threshold [CDT] at the vertex-level) for multiple comparisons were superimposed on clusters identified with analyses using the revised LIWC emotion word dictionary (see Results section). None of the trends using the standard LIWC emotion word dictionary survived family-wise error correction for multiple comparisons at the cluster-level (FWE p< .05, two-tailed; whole-brain).

For additional analyses pertaining to the comparison of the revised versus standard LIWC emotion word dictionary, cortical thickness change scores of significant clusters (already controlled for global cortical thickness change at each time point (Valk et al., 2017)) after the Perspective Module derived from the analysis with the revised dictionary were extracted and examined using linear mixed models (LMMs) in SPSS (IBM Corp, 2013). Cortical thickness change scores were used as dependent variable, emotional word use change scores using the standard LIWC emotion word dictionary as independent variable, and analyses were additionally controlled for mean-centered age and sex. Results showed that negative emotional word use change scores derived from the standard LIWC emotion word dictionary were positively associated with cortical thickness change scores extracted from the left pars orbitals (*b* = .0035, *t*(106) = 3.590, *p* = .001, 95% CI [.0016, .0055]) and the right dlLPFC (*b* = .0034, *t(*106) = 3.353, *p* = .001, 95% CI [.0014, .0055]) clusters using the revised LIWC emotion word dictionary. In addition, overall emotional word use change scores derived from the standard LIWC emotion word dictionary were marginally associated with extracted cortical thickness change from the right mPFC-dlPFC (*b* = .0009, *t*(106) = 1.850, *p* = .067, 95% CI [-.0001, .0019]) identified with the revised LIWC emotion word dictionary.

**S3. Robustness of Relationship between Cortical Thickness Change and Emotional Word Use Change**

Post-hoc analyses showed that the relationship between increase in overall emotional word use and increase in cortical thickness in the right mPFC-dlPFC cluster is significant for both TC1 (*b* = .0031, *t*(47) = 2.426, *p* = .019, 95% CI [.0005, .0057]) and TC2 (*b* = .0021, *t*(55) = 2.918, *p* = .005, 95% CI [.0007, .0036]) separately. The relationship between increase in negative emotional word use and increase in cortical thickness in the left pars orbitalis is also significant for TC2 alone (*b* = .0043, *t*(55) = 3.397, *p* = .001, 95% CI [.0018, .0068]), and marginally significant for TC1 alone (*b* = .0034, *t*(47) = 1.975, *p* = .054, 95% CI [-.0001, .0068]). Finally, the relationship between increase in negative emotional word use and increase in cortical thickness in the right dlPFC cluster is again significant for both TC1 (*b* = .0066, *t*(47) = 3.777, *p* < .001, 95% CI [.0031, .0101]) and TC2 (*b* = .0029, *t*(55) = 2.468, *p* = .017, 95% CI [.0005, .0052]) separately.

**S4. Confirmatory Analysis of the Relationship between Cortical Thickness Change and Emotional Word Use Change**


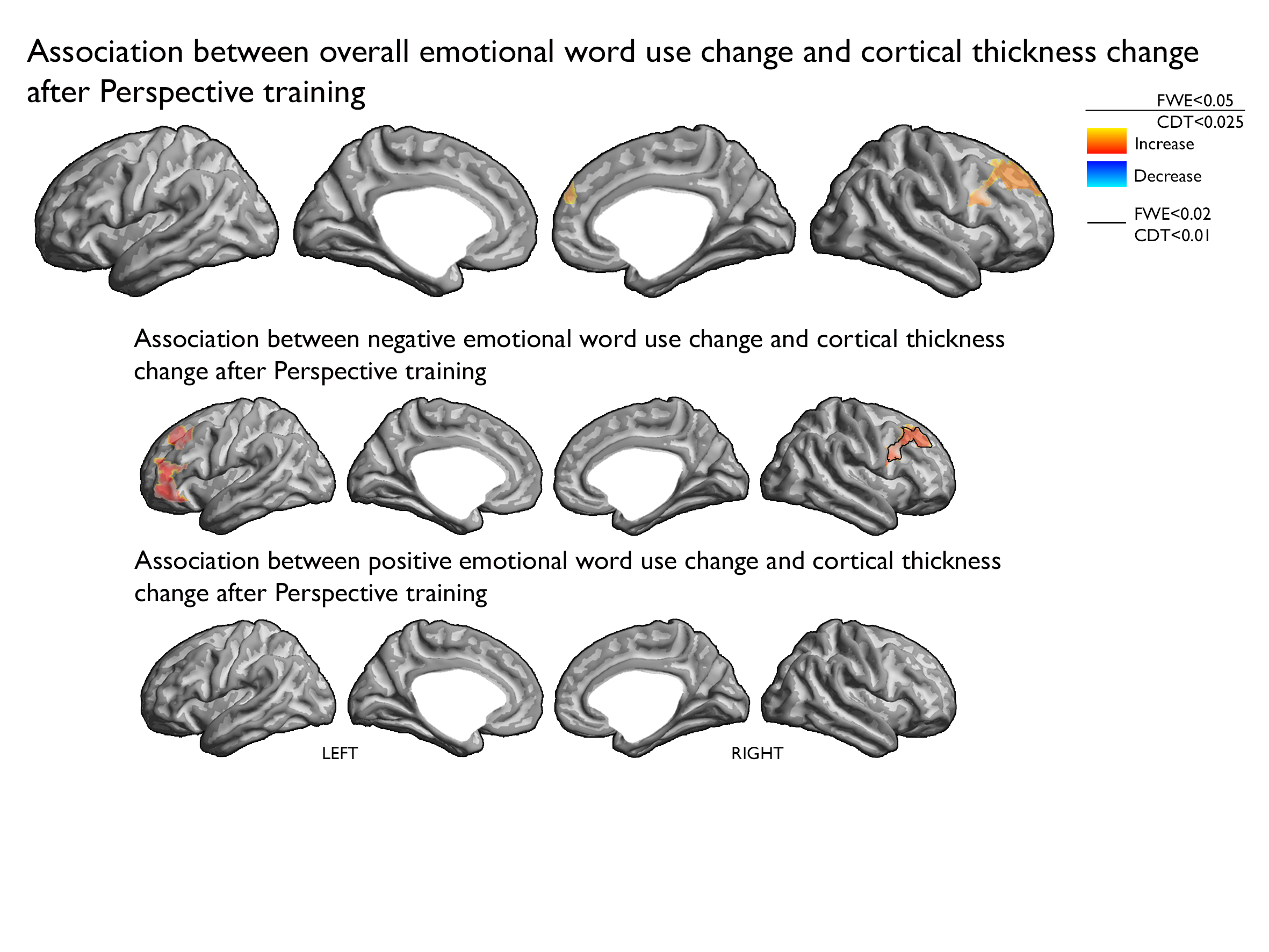


*Figure S4.* Relationship between increase in cortical thickness and increase in negative emotional word use after training in the Perspective Module in the right dlPFC. Findings with a more stringent cluster-determining threshold (CDT) of p< .01 and FWE of p< .02 (two-tailed, black outlines) are superimposed on the main findings reported in the manuscript.

**References**

IBM, Corp. (Released 2013). IBM SPSS Statistics for Windows, Version 22.0. In. Armonk, NY: IBM Corp.

Lumma, A. L., Böckler, A., Vrtička, P., & Singer, T. (2017). Who am I? Differential effects

of three contemplative mental trainings on emotional word use in self-descriptions.

*Self and Identity*, 1-22.

Valk, S. L., Bernhardt, B. C., Trautwein, F. M., Böckler, A., Kanske, P., Guizard, N., …

Singer, T. (2017). Structural plasticity of the social brain: Differential change after socio-affective and cognitive mental training. *Science Advances*, *3*(10), e1700489. https://doi.org/10.1126/sciadv.1700489
